# Cardiac arrest provoked by itraconazole and amiodarone interaction: a case report

**DOI:** 10.1186/1752-1947-5-333

**Published:** 2011-07-29

**Authors:** Angeliki M Tsimogianni, Ilias Andrianakis, Alex Betrosian, Emmanouil Douzinas

**Affiliations:** 1 Third Intensive Care Department, Evgenidion Hospital, 20 Papadiamantopoulou Street, 11528, Athens, Greece

## Abstract

**Introduction:**

Azoles, and specifically itraconazole, are often prescribed for the treatment of fungal diseases or empirically for persistent sepsis in patients who are neutropenic or in intensive care. Occasional cardiovascular adverse events have been associated with itraconazole use, and are usually attributed to the interaction of itraconazole with cisapride, terfenadine or digoxin. Its interaction with amiodarone has not been previously described.

**Case presentation:**

A 65-year-old Caucasian man was admitted to the Intensive Care Unit at our facility for an extensive ischemic stroke associated with atrial fibrillation. Due to rapid ventricular response he was started on intravenous amiodarone and few days later itraconazole was also prescribed for presumed candidemia. After receiving the first dose our patient became profoundly hypotensive but responded rapidly to fluids and adrenaline. Then, two months later, itraconazole was again prescribed for confirmed fungemia. After receiving the first dose via a central venous catheter our patient became hypotensive and subsequently arrested. He was resuscitated successfully, and as no other cause was identified the arrest was attributed to septic shock and his antifungal treatment was changed to caspofungin. When sensitivity test results became available, antifungal treatment was down-staged to itraconazole and immediately after drug administration our patient suffered another arrest and was once again resuscitated successfully. This time the arrest was related to itraconazole, which was discontinued, and from then on our patient remained stable until his discharge to our neurology ward.

**Conclusions:**

Itraconazole and amiodarone coadministration can lead to serious cardiovascular adverse events in patients who are critically ill. Intensivists, pharmacists and medical physicians should be aware of the interaction of these two commonly used drugs.

## Introduction

Itraconazole has been widely used in the treatment of fungal diseases including chronic necrotizing pulmonary aspergillosis [1]. Patients in intensive care units with persistent high temperature despite broad-spectrum antibiotic treatment should be considered for empirical antifungal treatment due to the high incidence of candidemia and high mortality rates reported [2]. Azoles, fluconazole and itraconazole are the most justified options in this situation [3]. Itraconazole use has been associated with occasional adverse cardiovascular events, including congestive heart failure [4], hypertension, premature ventricular contractions or other dysrhythmias [5,6], and ventricular fibrillation [7]. These adverse events have been considered uncommon and are most often attributed to interaction of itraconazole with cisapride, terfenadine or digoxin [8].

In this study, we describe the case of a patient with ischemic stroke who had one hypotensive episode and two cardiac arrests after administration of intravenous itraconazole. To the best of our knowledge this is the first case reported of cardiac arrest due to interaction of itraconazole and amiodarone.

## Case report

A 65-year-old Caucasian man with history of hypertension was admitted to our neurology department with sudden loss of consciousness associated with right-sided hemiplegia. An electrocardiogram (ECG) showed atrial fibrillation (AF) with rapid ventricular response; his echocardiogram ejection fraction of 60% with no wall hypokinesia and computed tomography (CT) results confirmed ischemic changes in the area of distribution of the left anterior cerebral artery. He was started on intravenous amiodarone (Angoron; Sanofi Aventis) for rate control. Over the next few hours our patient's level of consciousness deteriorated and he required intubation and transfer to our intensive care unit. A few days later, he developed septic shock complicated with acute renal failure, and in addition to broad-spectrum antibiotic treatment empirical antifungal treatment with itraconazole (Micronazole; Pharma Line) was also prescribed. Soon after administration of the first dose our patient became profoundly hypotensive (blood pressure 50/30 mmHg) without obvious ECG changes. He responded to fluids, bolus and adrenaline infusion and within a couple of hours vasoactive drugs were discontinued. This hypotensive episode was considered septic, as no other apparent cause was identified.

Over the next few days, while our patient was stabilized and his renal function improved he developed anisocoria due to a new ischemic stroke on the opposite side, complicated with brain edema. He was treated conservatively and his level of consciousness progressively improved; he was weaned off the ventilator but due to the presence of a tracheotomy and intermittent septic episodes his stay in the intensive care unit was prolonged. Two months after his intensive care unit admission, fungi were isolated on blood cultures and our patient was started on itraconazole treatment, awaiting sensitivity test results. At that point, he had a high temperature but was breathing spontaneously and was hemodynamically stable. He was also receiving oral amiodarone 200 mg/day. After receiving the first dose of 200 mg itraconazole through a central venous catheter, our patient's blood pressure dropped suddenly from 150/90 mmHg to 50/30 mmHg and he subsequently lost consciousness and output; his ECG was compatible with pulseless electrical activity, with a rate of approximately 55 beats/minute. Our patient was connected to the ventilator again, cardiac massage was performed for three minutes and 2 mg of adrenaline was administered until the return of spontaneous circulation. Subsequently, noradrenaline infusion at a rate of 150 μg/minute was started, a dose that was progressively reduced and discontinued. His arterial blood gases, electrolytes and troponin immediately after resuscitation are shown in Table 1. His ECG after the arrest showed atrial fibrillation (AF), 100 bpm with prolonged corrected QT interval, with no other changes. A few hours later his QT interval returned to normal. Our patient's level of consciousness did not show any deterioration and results of a follow-up brain CT scan were slightly improved compared to the results of the previous scan. This episode was eventually attributed to sepsis from fungemia and the antifungal treatment was changed to caspofungin 50 mg (Cancidas; Merck) awaiting sensitivity test results. All medications administered to our patient on the day of the arrest are presented in Table 2. Five days later, our patient's blood culture results became available. *Candida parapsilosis *was isolated, sensitive to fluconazole and itraconazole, and antifungal treatment was down-staged to itraconazole. Due to an uncontrolled heart rate he also received a 300 mg of bolus amiodarone. A few minutes after receiving the itraconazole (200 mg) he suffered another pulseless electrical activity arrest; this time cardiac massage was performed for 10 minutes, and bolus adrenaline and atropine were administered until he was resuscitated successfully. His arterial blood gases, electrolytes and troponin immediately and six hours after resuscitation were again unremarkable. This time it became clear that itraconazole was related to the arrests and was discontinued. From then on, our patient remained stable and 15 days later was discharged back to our neurology ward.

**Table 1:** Routine tests after arrest

Test and parameters	Settings
Ventilator settings:	

Mode	Pressure regulated volume control

Tidal volume	600 mL

Frequency	18 breaths/minute

Positive end-expiratory pressure	5 mmHg

FiO_2_	100%

Arterial blood gases:	

PaO_2_	448 mmHg

PaCO_2_	15 mmHg

pH	7.37

HCO_3_	10 mm/L

Electrolytes:	

Na	142 mm/L

K	4.7 mmol/L

Lactate	5.7 mmol/L

Hematocrit	26.3%

Troponin	<0.05 ng/mL

Electrocardiogram	Atrial fibrillation, prolonged corrected QT interval, no ischemic changes

Pa = partial pressure.

**Table 2:** List of medications taken by our patient the day of the first cardiac arrest

Medication	Dose	Route of administration
Meropenem (Meronem)	1 g × 3	Intravenously

Human albumin 25% (Plasbumin)	50 mL × 2	Intravenously

Furosemide (Lasix)	20 mg × 2	Intravenously

Tigecycline (Tygacil)	50 mg × 2	Intravenously

Iron (Anemifer)	100 mg × 1	Intravenously

Pantoprazole (Pantosec)	40 mg × 2	Intravenously

Fondaparinux sodium (Arixtra)	2.5 mg × 1	Subcutaneously

Epoetin zeta (Retacrit)	40,000 IU^† ^once weekly	Subcutaneously

Amiodarone (Angoron)	200 mg × 1	Levin

*Saccharomyces boulardii *(Ultra Levure)	Two caps × 3	Levin

## Discussion

We present the case of a 65-year-old man with ischemic stroke who collapsed once and arrested twice after itraconazole infusion through a central venous catheter while receiving amiodarone for AF. The first and second time, the episodes were attributed to sepsis and that is why the medication was administered a third time. Our patient was resuscitated rapidly after these episodes and all alternative causes for the arrests were excluded. Our patient was followed up for 15 days after the last episode, was stable, and was eventually discharged back to our neurology ward. According to the Naranjo scale [9] this adverse reaction is graded as 9, which makes the association of itraconazole with the arrest definite (Table 3). A challenge test was not performed for ethical reasons.

**Table 3:** Naranjo scale

Questionnaire question and answer scoring	Answer/score
1. Are there previous conclusive reports on this reaction?	No (0)

Yes (+1); no (0); do not know or not done (0)	

2. Did the adverse event appear after the suspected drug was given?	Yes (+2)

Yes (+2); no (-1); do not know or not done (0)	

3. Did the adverse reaction improve when the drug was discontinued or a specific antagonist was given?	Yes (+1)

Yes (+1); no (0); do not know or not done (0)	

4. Did the adverse reaction appear when the drug was readministered?	Yes (+2)

Yes (+2); no (-2); do not know or not done (0)	

5. Are there alternative causes that could have caused the reaction?	No (+2)

Yes (-1); no (+2); do not know or not done (0)	

6. Did the reaction reappear when a placebo was given?	No (+1)

Yes (-1); no (+1); do not know or not done (0)	

7. Was the drug detected in any body fluid in toxic concentrations?	No (0)

Yes (+1); no (0); do not know or not done (0)	

8. Was the reaction more severe when the dose was increased, or less severe when the dose was decreased?	Yes (+1)

Yes (+1); no (0); do not know or not done (0)	

9. Did the patient have a similar reaction to the same or similar drugs in any previous exposure?	No (0)

Yes (+1); no (0); do not know or not done (0)	

10. Was the adverse event confirmed by any objective evidence?	No (0)

Yes (+1); no (0); do not know or not done (0)	

Scoring:	

>9 = definite adverse drug reaction (ADR), 5-8 = probable ADR, 1-4 = possible ADR, 0 = doubtful ADR	9 = definite ADR

In the literature [4-7], itraconazole use has been associated with occasional adverse cardiovascular events including congestive heart failure, hypertension and various dysrhythmias, most often in patients with pre-existing cardiac disease [6]. These adverse events have been considered uncommon and most times attributed to interaction of itraconazole with cisapride, terfenadine, digoxin or methadone [8]. The possible mechanisms for these adverse events are the negative inotropic effect of itraconazole *per se *and the prolongation of the QT interval leading occasionally to torsades de pointes, as itraconazole may increase plasma levels of the above medications (which prolong the QT interval). The rapid intravenous administration, insufficient dilution and drug vehicle have been associated with the dysrhythmias caused by imidazole antifungals. Our patient was concomitantly receiving amiodarone, the first and third time intravenously and the second time orally, which can prolong the QT interval but his ECG in the intensive care unit was monitored continuously and torsades de pointes did not occur. During both arrests his ECGs were compatible with pulseless electrical activity, slightly bradycardic and the first event that occurred was the sudden drop in blood pressure. Another explanation could be an anaphylactic-type reaction causing vasodilation and shock. We believe the most likely explanation for the arrests is the direct myocardial depression of the drug *per se *in combination with the negative inotropic [7] and chronotropic effect of amiodarone, enhanced by its increased plasma levels as a result of its reduced liver metabolism caused by itraconazole. Regardless, the rapid infusion though a central venous catheter and the interaction with amiodarone seems to have contributed to the arrest.

Making an appraisal of our patient management, we could first criticize that intravenous itraconazole was prescribed to our patient while he was on hemodiafiltration and had a creatinine clearance below 30 mL/minute. In the medical information for the drug, it is suggested the oral formulation be used for a creatinine clearance lower than 30 mL/minute due to possible accumulation of cyclodextrin. In our defense this is common practice in intensive care units; intravenous formulations are always preferred due to impaired absorption of drugs from the gastrointestinal tract in patients who are critically ill. Moreover, other medical sources only suggest halving the dose [10] for clearance <10 mL/minute. In subsequent administrations of the medication our patient's clearance was above 50 mL/minute. Finally, imidazole agents are the preferred treatment choices for the empirical treatment of fungal infections in intensive care units [2].

The limitations of our case report are first of all the difficulty in confirming the association of the drug with the arrest by an objective measurement. In our defense, this is common in adverse drug reactions, the association with a specific drug can be assessed with the Naranjo scale and according to this scale the cardiac arrest after itraconazole infusion was a definite adverse drug reaction [9]. More importantly, the mechanism of the arrest is not fully understood. Further studies are required to investigate the cardiologic side effects of itraconazole and the possible interaction of itraconazole and amiodarone, a commonly used antiarrhythmic drug in intensive care units.

## Conclusions

In this article, we present the case of a patient who had one hypotensive episode and two cardiac arrests after concomitant administration of itraconazole and amiodarone. To the best of our knowledge this considerable side effect has not been described in the literature previously. Intensivists, pharmacists and medical physicians of various specialties such as cardiologists, oncologists and hematologists, should be aware of this possible interaction with deleterious results. Patients receiving medications that can prolong the QT interval or with pre-existing cardiac disease should be administered the intravenous formulation of this medication with caution, especially if given through a central venous catheter.

## Consent

Written informed consent was obtained from the patient's next-of-kin for publication of this case report and any accompanying images. A copy of the written consent is available for review by the Editor-in-Chief of this journal.

## Competing interests

The authors declare that they have no competing interests.

## Authors' contributions

AMT resuscitated our patient from both arrests, identified the association of the arrest with the drug, had a significant contribution in acquisition and interpretation of patient data, a major role in drafting the manuscript and read and approved the final manuscript. IA had a leading role in drafting and revising the manuscript and has read and approved the version to be published. AB had the initial concept of presenting this case report, contributed significantly to the interpretation of our patient's data and revision of the manuscript, and read and approved the version of the manuscript to be published. ED read and approved the final version of the manuscript.
